# Characterization of steel lined with multilayer micro/nano-polymeric composites

**DOI:** 10.1038/s41598-022-22084-5

**Published:** 2022-11-10

**Authors:** M. Megahed, Kh. Abd El-Aziz, D. Saber

**Affiliations:** 1 Department of Mechanical Design and Production Engineering, Faculty of Engineering, Zagazig University, P.O. Box 44519, Zagazig, Egypt; 2 Materials Engineering Department, Faculty of Engineering, Zagazig University, Zagazig, 44519 Egypt; 3 Mechanical Engineering Department, College of Engineering, Taif University, Taif, Saudi Arabia; 4 Industrial Engineering Department, College of Engineering, Taif University, Taif, Saudi Arabia

**Keywords:** Engineering, Materials science, Nanoscience and technology

## Abstract

This work studied comparison of the mechanical and barrier resistance properties between different structures of three multilayers polymeric coating on each side of the steel coupons. Epoxy filled with 1 wt%, 2 wt%, and 3 wt% micron or nano-sized alumina (Al_2_O_3_) particles represented the coating layers to steel on both sides. Barrier resistance was performed by immersing the coated steel specimens in salt solution and in a citric acid medium. Adding alumina (Al_2_O_3_) particles in micron and nano size to epoxy coatings improved the barrier resistance, tensile, and hardness under dry and wet conditions as compared to pure epoxy coating. Further increases in Al_2_O_3_ micro/nanoparticles cause deterioration in tensile strength and barrier resistance. The steel lined with epoxy filled with 1 wt% Al_2_O_3_ nanoparticles has a maximum tensile strength of 299.5 MPa and 280.9 MPa under dry and wet conditions, respectively. However, the steel lined with epoxy filled with 1 wt% Al_2_O_3_ microparticles has a tensile strength of 296.5 MPa and 275.4 MPa under dry and wet conditions, respectively. Good properties were observed with stepwise graded micro/nanocomposite coatings. The steel lined with epoxy filled with 3 wt% Al_2_O_3_ nanoparticles has maximum hardness of 46 HV and 40 HV under dry and wet conditions, respectively.

## Introduction

The corrosion of metal is considered one of the vital issues for steel structures when these structures are subjected to corrosion^1^. Steel has high mechanical strength with low-cost fabrication. Consequently, it is utilized in drilling equipment, shipbuilding, and pipelines. In marines, corrosion results in 30% of the total failure and thus needed to be repaired or replaced parts. In a marine environment, the corrosion of steel is influenced by salinity and alkalinity^2^. Subsequently, the coating was performed on steel faces to avoid the corrosion of new or existing steel construction. The corrosion of steel attracted many research interests as it is costly, particularly in the oilfield and marine environments^3^. Recently, polymer composite liners to steel were used to decrease the diffusion of oxygen and moisture. The protective organic coating as an epoxy coating to metal is characterized by its excellent weather ability^4^. Protected epoxy coating has attracted great attention in wet environments due to its very good toughness, durability, and adhesion to metal substrates^1^. However, the highly cross-linking density and the barrier behavior of the epoxy coating can be undesirably affected when exposed to corrosion. The polymer coating weakening results in the creation of holes, and defects in the epoxy coating surface. During exposure to corrosive media, holes and defects become larger in width and depth. Holes are considered as conductive paths as the electrolyte diffuses in the polymeric coating^5^. Moreover, the protective coating fails by the cause of delamination which is the separation at the polymeric coating/metal interface^6^. The deterioration of polymeric coating decreases the barrier properties thus the mechanical properties of the polymeric coating^5^. Therefore, it is essential to enhance the properties of epoxy resin by replacing epoxy with epoxy composite coatings to achieve the requirements of real applications^4^.

Embedded inorganic fillers to epoxy coating are one of the methods to enhance the anti-corrosion characterization of organic polymeric coatings. Adding smaller filler particles in micron or nano size may improve the barrier properties of the introduced polymeric coating. Size, morphology, shape, and the weight percentage of the fillers greatly affect the intrinsic characteristics of composite^2^. Nanoparticles are considered a good water barrier and thus effectively obstruct water absorption improving the service life of metals^2^. Different nanomaterials are involved at various levels in the food industry having both positive and negative effects on human health. Alumina can also be present due to contamination or migration from other food contact materials such as processing machinery, utensils, and devices^7^. The coatings containing Al_2_O_3_ particles showed enhancement in scratch and abrasive resistance compared with that of the polymer coating. This enhancement in scratch and abrasive resistance is attributed to the dispersion hardening of Al_2_O_3_ nanoparticles in polymer coatings^8^. Enhancement in environmental impact can be attained by utilizing nanosized particulates in polymeric coating and eliminating the requirement for toxic solvents^9^. Nanoparticles embedded in polymeric coatings are well known for their outstanding physical, mechanical, and thermal properties^10,11^.

Ramezanzadeh and Attar^5^ investigated the corrosion resistance of the epoxy coating containing micron and nano-sized ZnO fillers. The specimens were submerged in 3.5 wt% NaCl solution. The corrosion resistance of the coupons was significantly decreased after immersion for 15 days. The corrosion resistance of the epoxy coating was enhanced as reinforced with nano-sized ZnO fillers. The results showed that the lowest reduction in cross-linking density and reducing the hardness of the polymeric coating submerged in 3.5 wt% NaCl solution was attained as the epoxy coating was reinforced with the 3.5 wt% nano ZnO particles. Moreover, the adhesion was also increased at 3.5 wt%. Furthermore, Anaki and Xavier^1^ studied the dispersibility of reinforcing epoxy coating on mild steel with 2 wt% of nano Al_2_O_3_. The resultant specimens have been submerged in a 3.5% NaCl solution. The improved anticorrosion performance was conducted by the modified nanocomposite coating as compared to epoxy coating. The reinforced epoxy coating resulted in good adhesive strength, increasing in hardness, tensile strength, and better corrosion resistance than epoxy coating. In addition, Golru et al.^12^ prepared epoxy/polyamide reinforced with 1, 2.5 and 3.5 wt% nano-alumina filler coted AA1050 substrate. The results showed that the nanofillers dispersed uniformly in the polymeric coating even when loading at high percentages. The polymeric coating corrosion resistance was more improved by increasing the weight percentage nanofillers.

In recent times, multilayered nanocomposites have gained great attention due to their required characteristics as microwave absorbing, mechanical properties, permittivity constructed on the interfaces between adjacent layers, and the synergistic impacts of fillers. Nevertheless, the application of multilayered micro/nanocomposite coatings has not been reported yet^4^. Al_2_O_3_ filler in micron size is commercially available and has a lower cost than Al_2_O_3_ in nano-size. So, the objective of the study is to develop multilayers of epoxy liners to steel filled with micro and nano Al_2_O_3_ particles with different percentages and differentiate between them. Three percentages of alumina micro and nanoparticles (1 wt%, 2 wt%, and 3 wt%) were introduced to epoxy with different configurations. The specimens were immersed in salt solution and in citric acid media. Barrier resistance and mechanical properties were investigated under dry and wet conditions.

## Experimental work

### Materials

Mild steel was used as a metal substrate supplied from Al Ezz-Dekheila Steel Company Alexandria. The steel sheets were cut to the required dimensions of the specimens by a laser machine. The specimens were polished in order to roughen the surface of the steel substrate. After polishing, the top and bottom side of the coupon's surface was cleaned with acetone before coating. Chemicals including sodium hydroxide, citric acid, and acetone were supplied by El Nasr Pharmaceutical Chemicals, Egypt. The coating is Epoxy resin (Kemapoxy RGL150) which is supplied by CNB Company, Egypt. The reinforcements are Al_2_O_3_ fillers in micron and nano size with a purity of about 99%. The size of microparticles and nanoparticles are 90 µm and 70 nm, respectively.

### Preparation of micro/nanocomposite protective films

**Figure 1:**
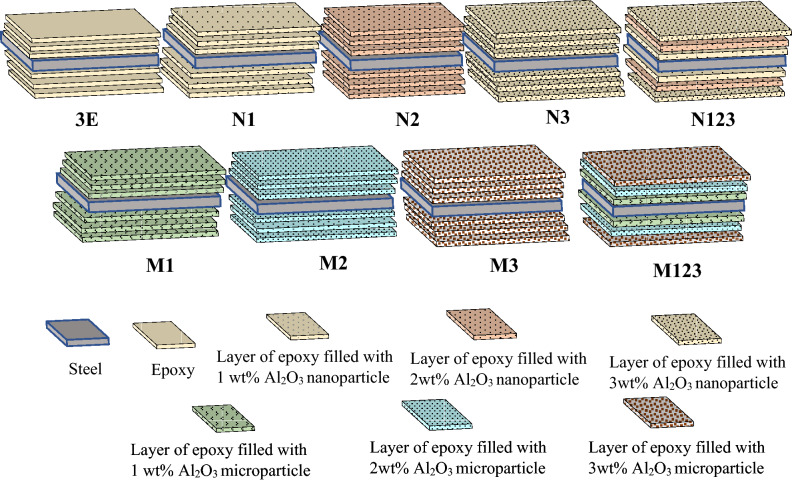
Construction of micro/nanocomposite coatings on steel substrate.

Epoxy protective films were made by adding hardener carefully to the epoxy and blended thoroughly with a ratio of 1:2 by mass of epoxy resin. The micro/nanocomposite protective films were performed as micro, or nanoparticles were added to the epoxy resin by sonication process. The sonication was performed with Hielscher ultrasonic processor UP 200 S. The sonication was conducted at 0.5 cycles per second on/off with an amplitude of 40% for 2 h as recommended by^13,14^. For epoxy resin protection from degradation, the mixture was cooled by placing it on an ice water bath during sonication^15^. Afterward, the blend and the hardener were mixed with the recommended ratio at a temperature of 25 °C. The protective layer was prepared on steel by a metallic roller to remove excess resin and reduce void content and any entrapped air bubbles. Painting on one side of the steel specimen is left for 24 h to cure. Subsequently, the second layer on the same side was constructed and left for a day to cure. and the same with the third layer. The same technique was done for the other three layers on the bottom face of the coupons. The final stepwise graded and non-graded micro/nanocomposite coatings on steel substrate were constructed as illustrated in Fig. 1.

### Experimental procedure

#### Tensile test

The tensile behavior of the steel coated with micro/nanocomposites coupons were tested according to ASTM D3039. The tensile test was achieved with a computerized universal testing machine (Jinan Test Machine WDW 100 kN). The cross-head speed was set at 2 mm/min and the stress–strain curve was recorded by a computer data acquisition system. All tests were performed at ambient temperature.

#### Hardness

The hardness was determined via the PCE-1000N Hardness instrument measured at ten different places of the micro/nanocomposite coated steel and the average value was taken.

#### Barrier properties

Some test coupons were immersed in salt solution and in citric acid solution to estimate the corrosion media of the steel lined with micro/nanocomposite. The salt solution was performed as 3.5% NaCl dissolved in water. Citric acid solution with a concentration of 2 N was prepared with double distilled water. Uptake tests were performed according to ASTM D5229/D5229M-14. The coated coupons were periodically withdrawn from the solutions, wiped dry, and weighed using an analytical balance of accuracy up to 10^−4^ g to monitor the weight change during the absorption process. The solution content M(t) absorbed by micro/nanocomposite protective coating was then calculated as the mass gain percentage referring to its initial weight (*w*_0_) as follows^16^:

1 $$M(t) = \left( {\frac{{w_{t} - w_{0} }}{{w_{0} }}} \right) \times 100$$

where *w*_t_ is the coupon mass after time t. Coated coupons were immersed up to 21 days.

## Results and discussions

### Hardness

**Figure 2:**
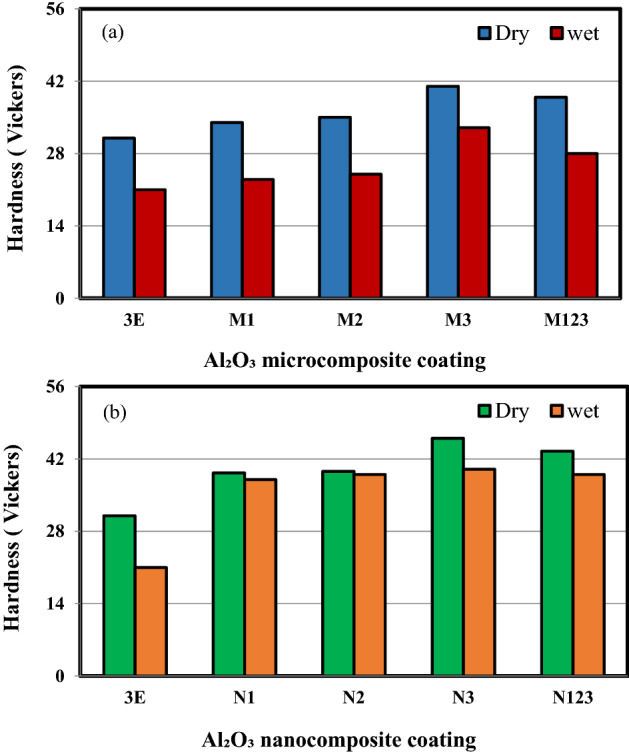
The hardness of multilayers epoxy coating to steel substrate filled with (**a**) Al_2_O_3_ microparticles (**b**) Al_2_O_3_ nanoparticles.

Figure 2a,b shows the hardness of multilayers epoxy coating to steel substrate filled with Al_2_O_3_ microparticles and Al_2_O_3_ nanoparticles under dry and wet condition, respectively. In wet conditions, the coated steel specimens were immersed in a salt solution for 35 days. The improvement in hardness in dry and wet conditions was attained in both sizes of Al_2_O_3_ particles as compared to pure epoxy coating. Moreover, as the weight percentage of the micron and nanoparticles increased, the hardness increased. This increase in hardness is due to increasing the Al_2_O_3_ particles content up to 3 wt% on the surface of coated steel specimens may be attributed to the high value of the hardness of ceramic particles as Al_2_O_3_ particles as compared to the hardness of the polymer. Furthermore, during measuring with the hardness indenter, the force results in the load applied increasing which in turn presses the epoxy causing the particles to touch each other giving more resistance to the force applied. As the weight percent of Al_2_O_3_ particles content increases, micro/nanoparticles fill in the gaps presented in the polymeric matrix as cracks and voids thus increasing hardness^17,18^. In addition, the hardness of the stepwise graded micro/nanocomposite coating gives high hardness as compared to composite liners filled with 1 wt% and 2 wt% micro/nano Al_2_O_3_ particles. This may be attributed to the higher percentage of micro/nano Al_2_O_3_ particles (3 wt%) on the outer surface of coated steel specimens, followed by 2 wt% Al_2_O_3_ particles then 1 wt% Al_2_O_3_ particles.

It can be depicted from Fig. 2a,b that the hardness deteriorated as the coated specimens were immersed in a salt solution. This decrease in the hardness value is mainly due to the seawater absorption which produced plasticization which is the softening and increase in the flexibility of the epoxy. Moreover, the seawater absorption caused damage if the interface between particle and matrix and also between the layers. Due to the uptake of seawater molecules by the coated specimens, the link between molecules of epoxy may disturb and the polymeric composite liners become so soft that the attachment between Al_2_O_3_ particles and epoxy got weakened. In addition, the epoxy got swelled due to the water absorption hence generating pressure to Al_2_O_3_ particles that results in pulling out of the particles from the epoxy forming micro-cracks inside the coated specimen. This reduces the hardness of the specimens in wet conditions as compared to the hardness of equivalent specimens in dry conditions^19^.

**Figure 3:**
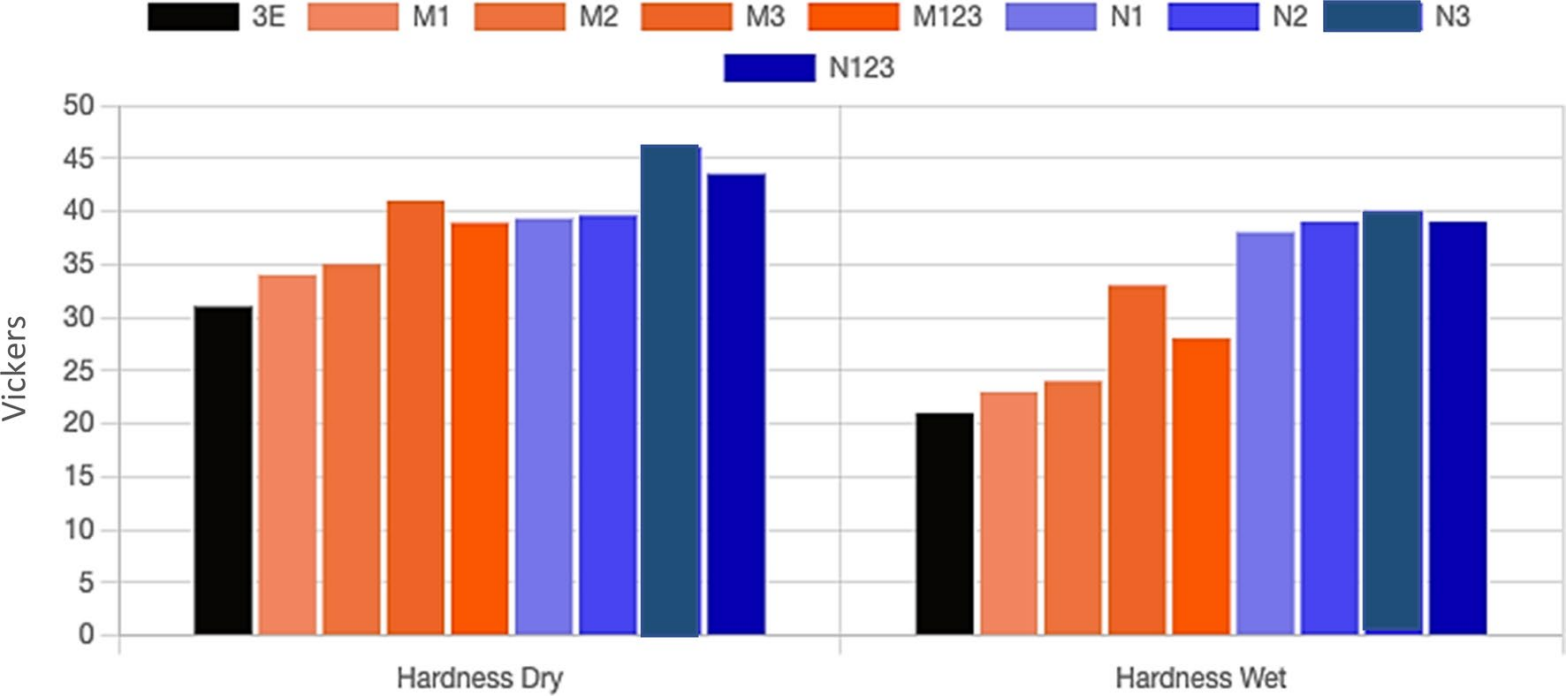
A comparison between the hardness values of microcomposite and nanocomposite coating under dry and wet conditions.

Figure 3 shows a comparison between the hardness values of microcomposite and nanocomposite coating under dry and wet conditions. It is clear from the figure that the highest hardness value was obtained with the addition of 3 wt% nanometer-sized Al_2_O_3_ particles either in dry or wet conditions. Followed by stepwise graded nanocomposite coating. This indicates the high effect of Al_2_O_3_ nanometer-sized particles in strengthening the epoxy matrix. It is attributed to the great surface area of Al_2_O_3_ nanoparticles compared to Al_2_O_3_ microparticles^20^. The specimen N3 exhibited the highest hardness value with an improvement of 48.4% and 90.48% in dry and wet conditions, respectively.

### Tensile properties

**Figure 4:**
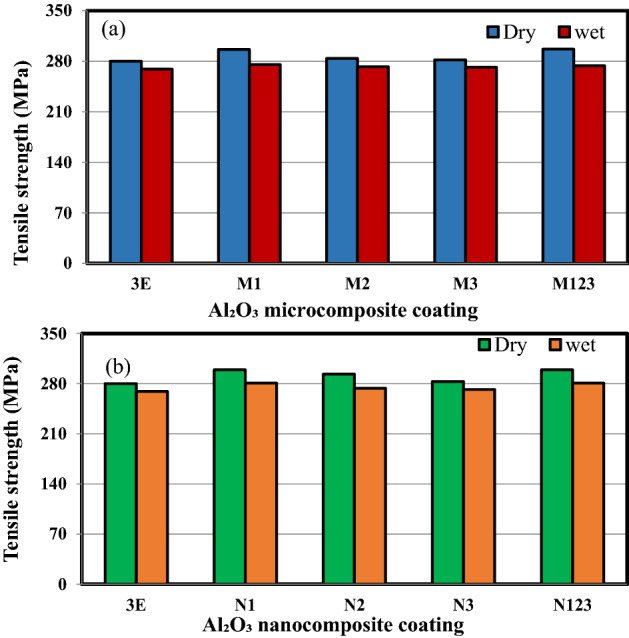
The tensile strength of multilayers epoxy coating to steel substrate filled with (**a**) Al_2_O_3_ microparticles (**b**) Al_2_O_3_ nanoparticles.

The tensile strength of multilayer epoxy coating to steel substrate filled with Al_2_O_3_ microparticles and Al_2_O_3_ nanoparticles under dry and wet conditions is shown in Fig. 4a,b, respectively. The results demonstrated that after immersion of all specimens in seawater, the tensile strengths deteriorated. Water absorption lowers the mechanical properties of steel coated with polymeric composites. The introduction of water molecules led to the change in the structure of the epoxy matrix and the interface between Al_2_O_3_ micro/nanoparticles and the epoxy matrix. Water that was introduced inside the layers of the coatings caused the interface to be damaged and thus polymer matrix cracked resulting in degrading the mechanical properties of the polymeric composite coating^21^. The debonding between coating layers and at the particle/matrix interface affected the stress transfer and thus the reinforcing effect of micro/nano Al_2_O_3_ particles on the epoxy matrix^22^. As the weight percentage of the micron and nano Al_2_O_3_ particles increased, the tensile strength decreased. Adding 1 wt% micro/nano Al_2_O_3_ particles gives the maximum enhancement of tensile strength in dry and wet conditions as compared to pure epoxy coating. Incorporation of small weight percentages of fillers leads to substantial improvement in mechanical properties of polymeric composite^23^. From Fig. 4a, it is clear that when adding the fillers with micron size to the epoxy with different weight percentages, the tensile strength was enhanced. The tensile strength of M1 was close to M123 which exhibited an improvement of 5.97% as compared to pure epoxy coating in dry conditions. The specimen M1 exhibited the highest improvement of 2.31% under wet conditions. However, the least improvement of 0.66% and 0.92% in tensile strength was obtained with specimen M3 under dry and wet conditions, respectively. Figure 4b shows that specimen N1 exhibited the maximum improvement in tensile strength of 6.92% and 4.33% under dry and wet conditions, respectively. The least improvement was obtained with specimen N3. The addition of a higher nanofiller weight percentage implies worse dispersions. The aggregations generally act as stress concentrators which in turn decreased the mechanical properties^24–29^.

**Figure 5:**
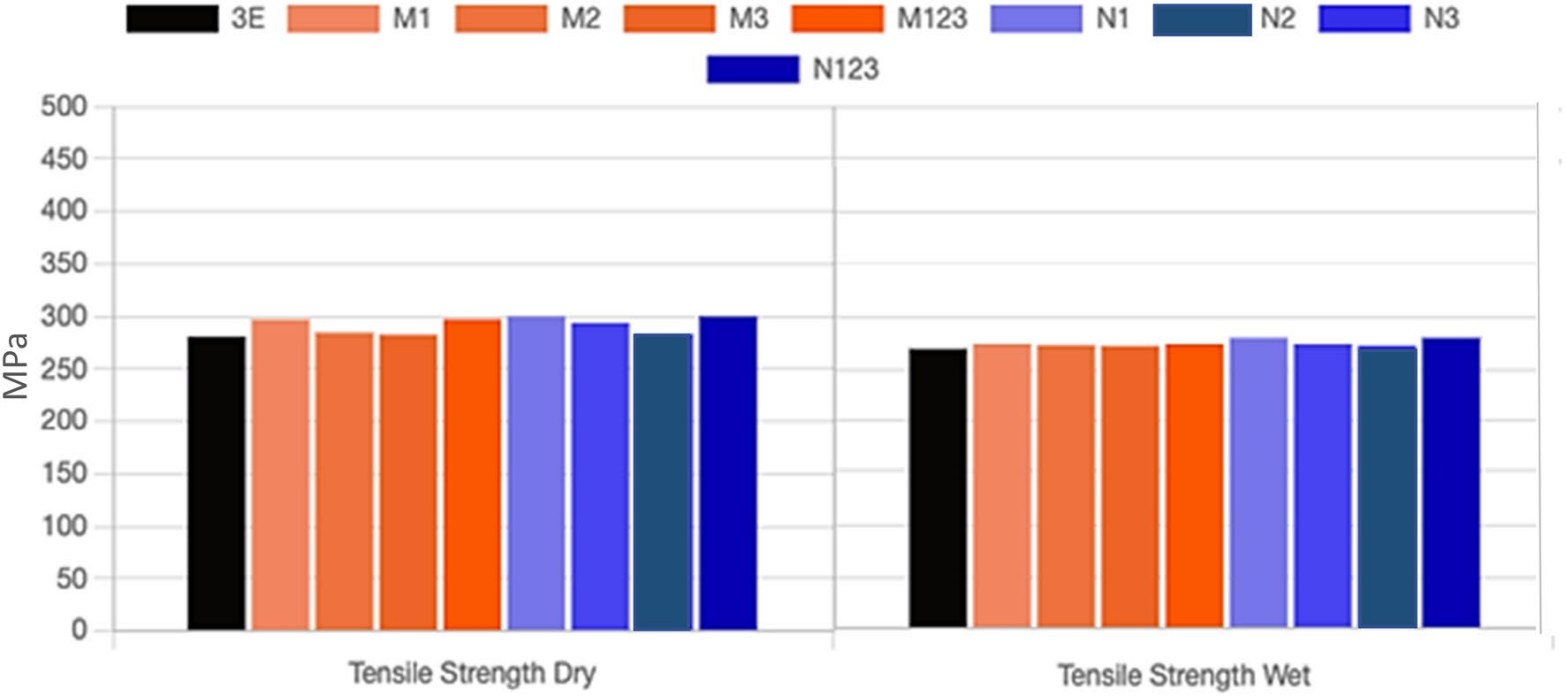
Tensile strength of micro/nanocomposite coating under dry and wet conditions.

Figure 5 shows a comparison between the tensile strength of microcomposite and nanocomposite coating under dry and wet conditions. The figure shows that the tensile strength deteriorated as the coated specimens were immersed in a salt solution. The water absorption primarily induces plasticization, decreasing the mechanical strength and rigidity of composite materials^24^. The specimen N1 exhibited the maximum improvement in tensile strength under dry and wet conditions, respectively. The least improvement was obtained with specimen M3. Both the stepwise graded steel lined with micro/nanocomposite are close to specimens coated with epoxy filled with 1 wt% Al_2_O_3_ micro/nanoparticles.

The mechanical properties of polymeric coating filled with particles depend on the filler size, filler/polymeric matrix interface adhesion, and filler content. For a given filler content, the polymeric composite strengths are enhanced by decreasing the filler size. Nanocomposite coatings such as N1, N2, N3, and N123 have a high total surface area than microcomposite coatings such as M1, M2, M3, and M123. Hence, the hardness and tensile strength are enhanced by increasing the total surface area of the reinforced particles with a further effective stress transfer mechanism. The filler/matrix interface's adhesion strengths control the load transfer between the constituents. Effective stress transfer is considered the most essential factor that contributes to the strength of the two constituents of the polymeric composite materials. For weak bonded fillers, the stress transfer at the filler/polymer interface is ineffective. Discontinuities occurred in the form of debonding because of poor adherence of filler to the polymeric matrix. Consequently, the filler cannot carry any load thus the polymeric composite strength reduces with increasing filler content. Nevertheless, for polymeric composite reinforced with well-bonded fillers, the addition of fillers to a polymeric matrix resulted in an increase in mechanical properties mainly for nanofillers with high surface areas^30^.

**Figure 6:**
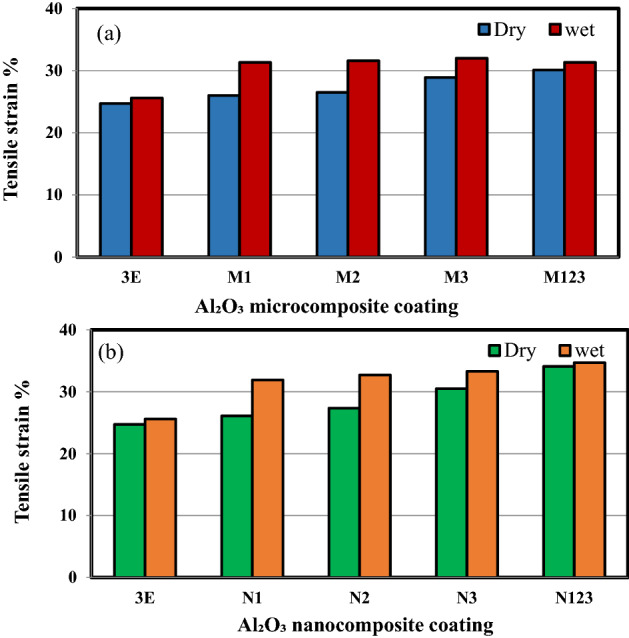
The tensile strain of multilayers epoxy coating to steel substrate filled with (**a**) Al_2_O_3_ microparticles (**b**) Al_2_O_3_ nanoparticles.

**Figure 7:**
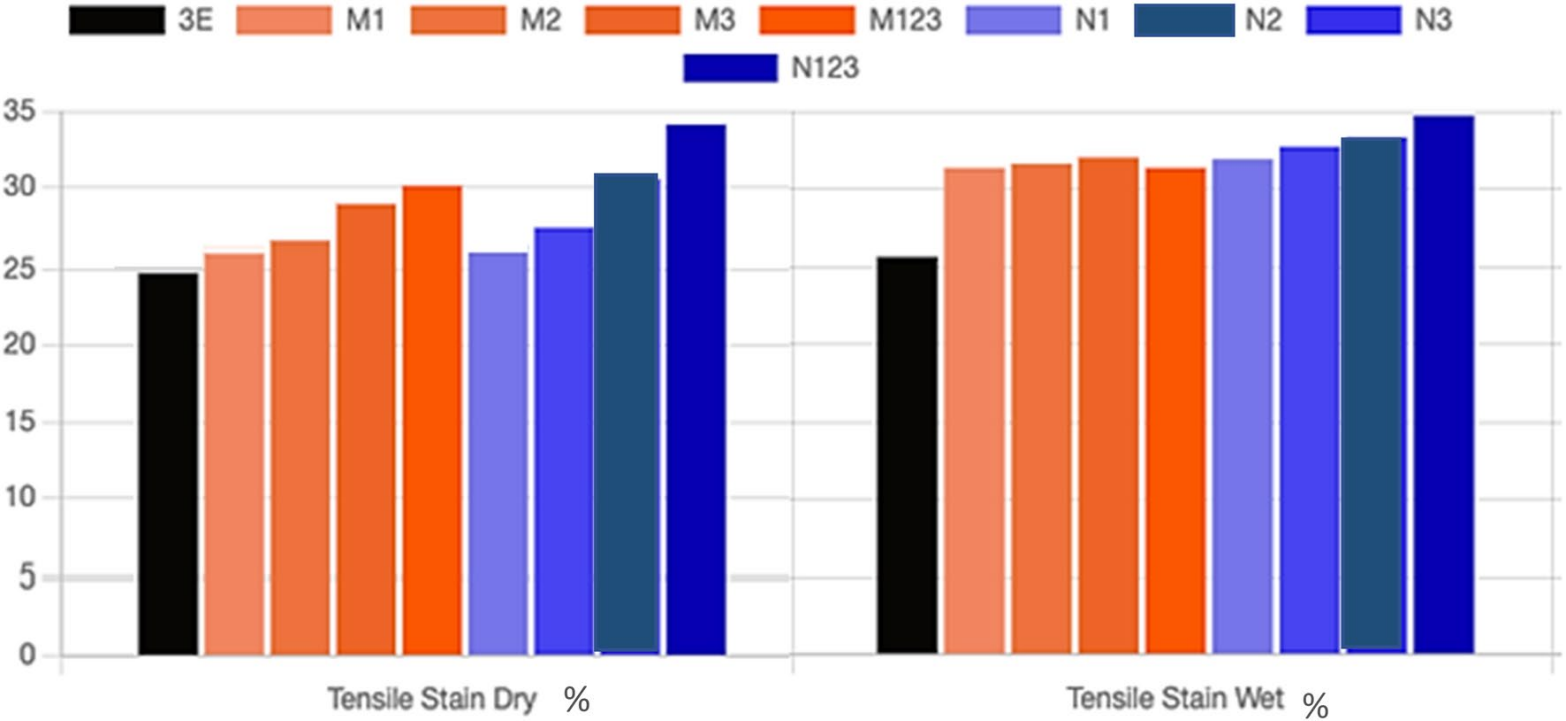
Tensile strain of micro/nanocomposite coating under dry and wet conditions.

Figure 6a,b shows the tensile strain of multilayer epoxy coating on a steel substrate filled with Al_2_O_3_ microparticles and nanoparticles. When adding the Al_2_O_3_ micro/nanoparticles to the epoxy, the tensile strain improved as compared to pure epoxy under dry and wet conditions. As the weight percentage of Al_2_O_3_ fillers increases, the tensile strain increases. Immersing the specimens in a salt solution leads to increasing the tensile strain of both sizes of Al_2_O_3_ particles as compared to pure epoxy coating. The ductility for both unfilled epoxy and Al_2_O_3_ filled micro/nanocomposite was enhanced as a result of water absorption. This can be attributed to the plasticization effect of water as the immersion time increased which can improve the ductility of the epoxy resin^31–33^. Figure 7 shows a comparison between the tensile strain of micro/nanocomposite coating under dry and wet conditions. The maximum improvement in tensile strain in the dry condition and wet conditions is obtained from N123 by 37.15% and 35.5, respectively. This is followed by an enhancement of 23.4% and 30% with N3 specimen in dry and wet conditions as compared to pure epoxy, respectively.

The high surface area of Al_2_O_3_ nanoparticles is the best attractive issue accordingly developing a large interface in a polymeric composite coating^20,34–37^. With adding 3 wt% Al_2_O_3_ nanoparticles to epoxy coating, the amount of Al_2_O_3_ nanoparticles is very high resulting in particle-to-particle interaction rather than the particle-to-epoxy interaction. Consequently, the Al_2_O_3_ particles begin to aggregate and form clusters that influence the Van der Waals interaction between the polymeric matrix chains reducing the cross-linking and increasing the void content. So, the resulting mechanical properties are therefore degraded^34,35^.

The fracture occurred in the steel coated with multilayers of filled epoxy is accompanied by a delamination between coating layers, matrix cracking and delamination between steel and coated layers. Delamination occurred at the interface between adjacent layers. Delamination occupies the most failure in polymeric composites as subjected to different kinds of testing. As the specimens loaded, further growth of interlayer delamination leads to final failure. Delamination is formed from interlaminar stresses being developed at the interfaces between adjacent layers. The fracture of filler/polymeric matrix interface stresses formed layer cracks that subsequently act as initiation places for delamination. Good interfacial bonding occurred between polymeric composite coating layers hindering the formation of delamination. Adding Al_2_O_3_ micro/nanoparticles to the epoxy matrix led to the development of a good filler/polymeric matrix interface hence reducing the delamination between coating layers and consequently increasing the mechanical properties^20^.

### Barrier properties

**Figure 8:**
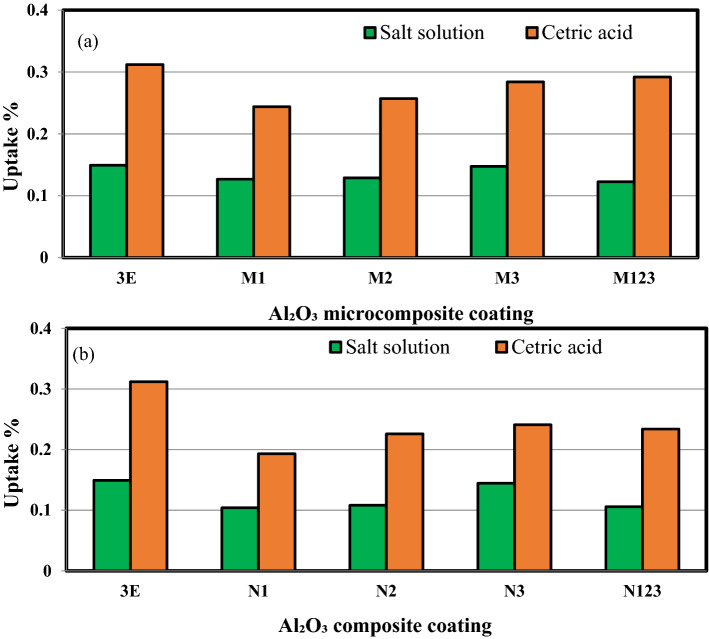
The barrier properties of multilayers epoxy coating to steel substrate filled with Al_2_O_3_ micro/nanoparticles immersed for 35 days in (**a**) salt solution (**b**) citric acid.

Figure 8a,b shows the barrier properties of multilayers epoxy coating to steel substrate filled with Al_2_O_3_ micro/nanoparticles immersed in salt solution and citric acid for 35 days. It is clear from the figures that the uptake% is significantly higher than that of seawater for both Al_2_O_3_ micro and nanocomposite coatings.

**Figure 9:**
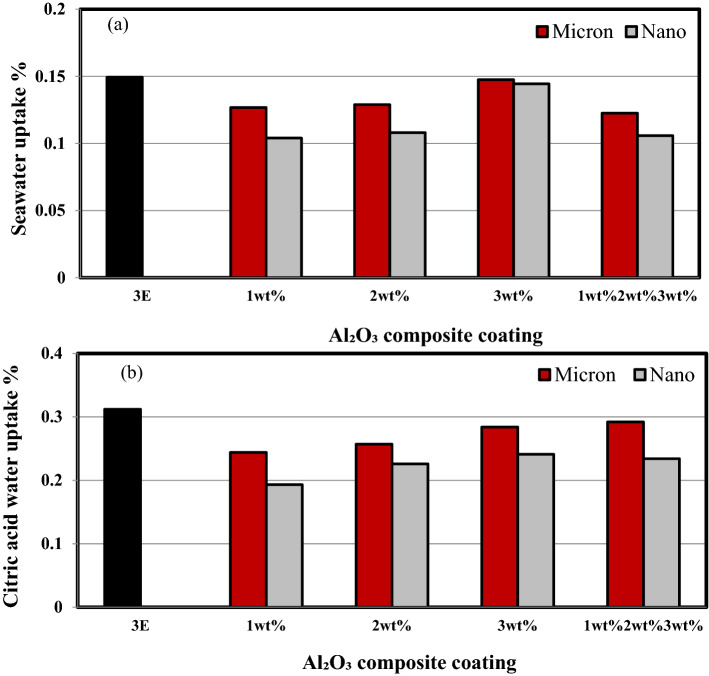
The barrier properties of multilayers epoxy coating to steel substrate filled with (**a**) Al_2_O_3_ microparticles (**b**) Al_2_O_3_ nanoparticles.

Figure 9a,b shows the barrier properties of multilayers epoxy coating to steel substrate filled with Al_2_O_3_ micro/nanoparticles. The uptake for both solutions was decreased with decreasing the size of the Al_2_O_3_ particles. The rate of water absorption increases with the increase in Al_2_O_3_ micro/nanoparticles content. This may be attributed to the increase in voids that formed with the presence of high content of Al_2_O_3_ micro/nanoparticles content and also due to the poor Al_2_O_3_/epoxy adhesion resulted in microcrack formation due to Al_2_O_3_ micro/nanoparticles agglomeration formed in the polymeric matrix. Furthermore, under the effect of seawater, the Al_2_O_3_ micro/nanoparticles tend to leave their places forming voids that are filled with seawater due to the capillary effect^22,36,37^. The decrease in water uptake rate is higher for epoxy coating filled with Al_2_O_3_ nanoparticles. This may be attributed to the good barrier properties of Al_2_O_3_ nanoparticles that form tortuous paths that hindered the seawater movement so reducing the water absorption rate^38^. Improved corrosion and mechanical properties were observed by filling the cracks presented in epoxy coats. The nanoparticles act as a strong barrier that can avoid penetration of aggressive ions to the steel surface^2^. Consequently, nanoparticles with very fine grain size and high boundary volume offer enhanced barrier properties as compared to conventional fillers^9^.

**Figure 10:**
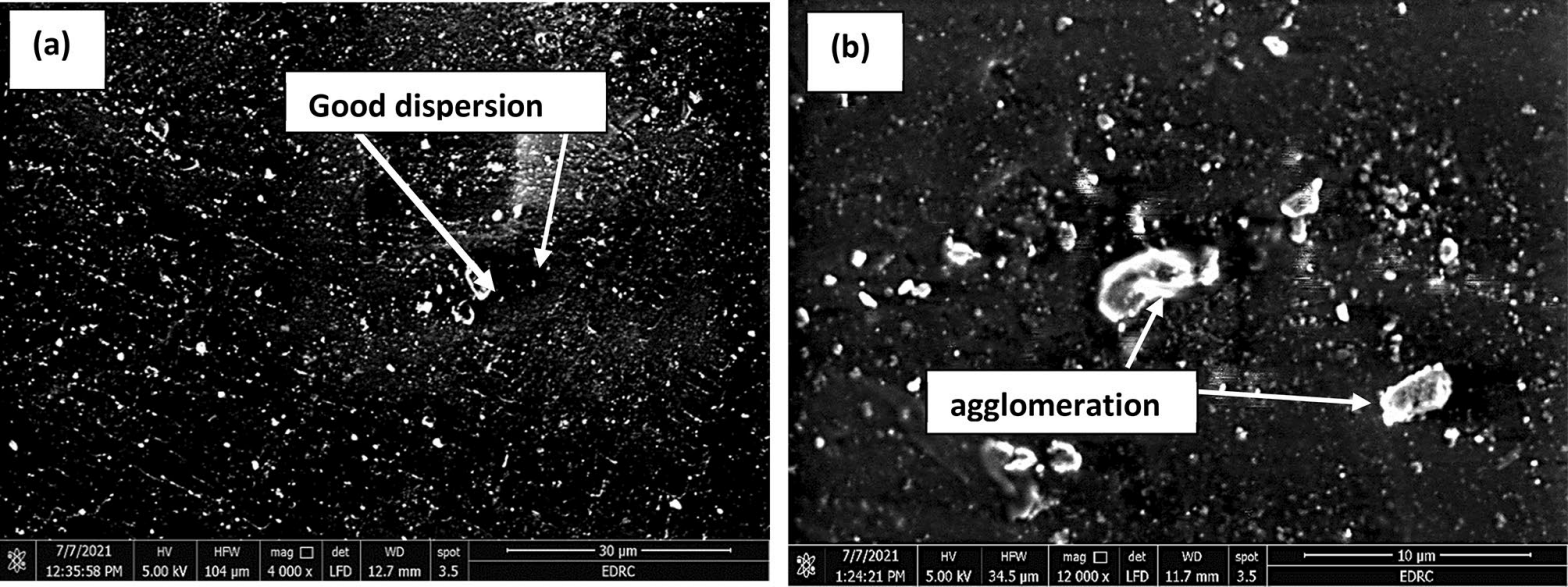
SEM showing the dispersion of alumina nanoparticles in (**a**) N1 and (**b**) N3.

The least uptake % was attained with steel coated with 1 wt% Al_2_O_3_ nanoparticles. This can be attributed to the good dispersion of low weight percentage (1 wt%) of Al_2_O_3_ nanoparticles as shown in Fig. 10a. Better properties can be accomplished when good dispersion and distribution of nanofillers are attained in the polymeric composites^39^. The inclusion of small weight percentages of nanofillers indicated substantial improvement in properties^23,40^.

It is observed that with a further increase in Al_2_O_3_ micro/nanoparticles to the epoxy, the uptake percentage increased. This may be attributed to the presence of agglomeration caused by the addition of more Al_2_O_3_ particles to the epoxy that helps in more absorption of water as shown in Fig. 10b. Therefore, the bigger the free volume of cured epoxy and the clearance between Al_2_O_3_ particles and epoxy resin, the more water permeable for the micro/nanocomposites are and the worse barrier properties can be attained. Furthermore, extra free volume presented at the interface also assisted the water permeability inside micro/nanocomposites. Seawater is more likely to diffuse along the epoxy/Al_2_O_3_ interface and destroy the interfacial bonding rather than diffuse through the epoxy matrix. So, as the free volume increased, water permeability increased^41^. Aggregation possesses rise to lower surface interactions of Al_2_O_3_-epoxy and higher stress concentration. This lead to lower the mechanical and barrier properties of the composites filled with nanofillers. However, a smaller aggregate size resulted in highly improved mechanical properties^42^. The uniform dispersion of Al_2_O_3_ nanoparticles verified more surface area of nanoparticles in the epoxy matrix. This increases the exposed surface area of Al_2_O_3_ nanoparticles to epoxy molecules resulting in cross-linking between Al_2_O_3_ nanoparticles and epoxy coating. This cross-linking enabled transferring the stress from epoxy to Al_2_O_3_ nanoparticles. The high strength of Al_2_O_3_ nanoparticles made them effective to carry extra loads when introduced to the polymeric matrix^25,43^.

**Figure 11:**
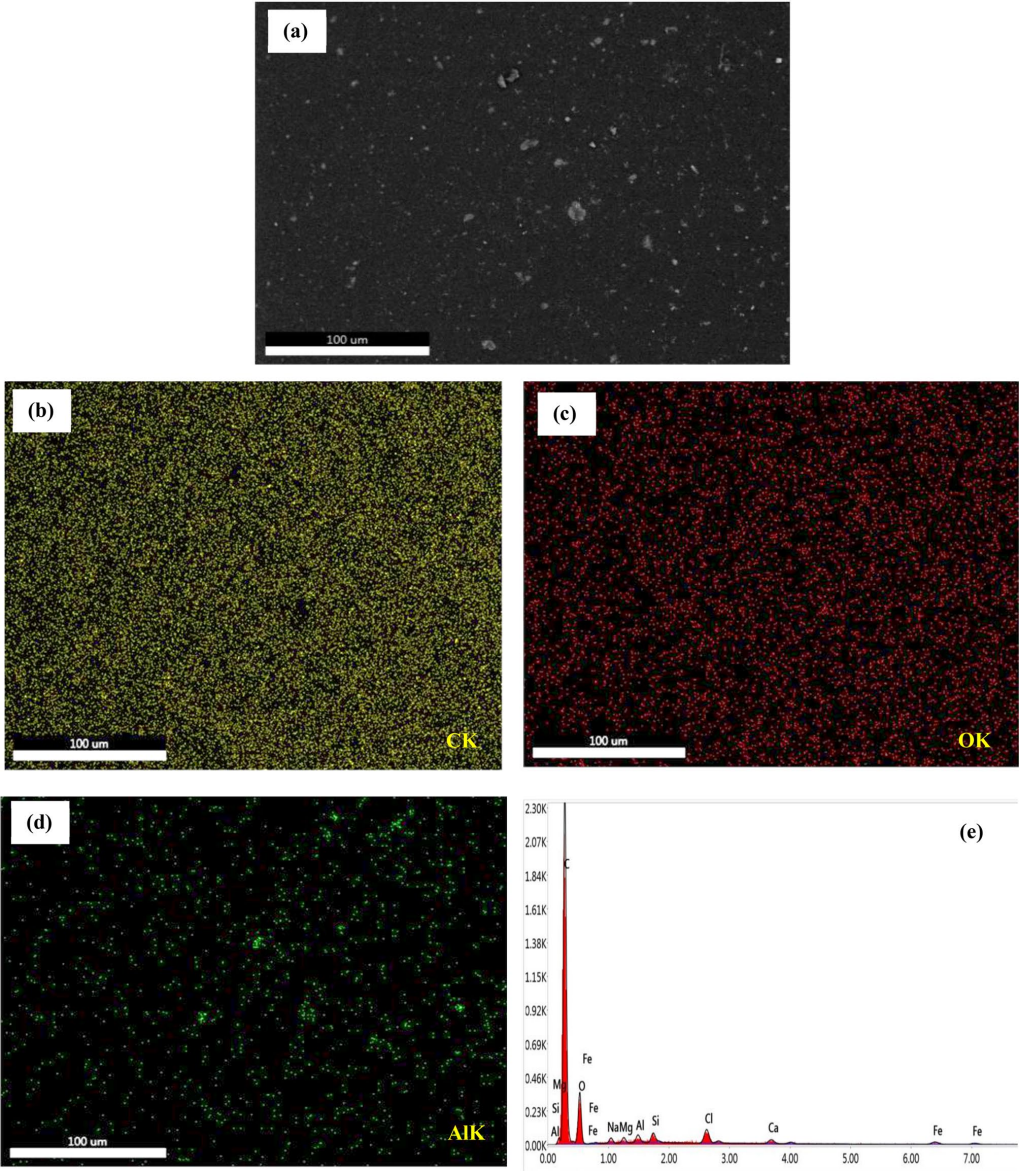
(**a**) FE-SEM of stepwise graded nanocomposite coating N123, (**b**)–(**d**) elemental map, (**e**) EDX spectrum.

The stepwise graded nanocomposite coating (N123) possess good barrier and mechanical properties. In order to determine the distribution of Al_2_O_3_ nanoparticles in the composites, surface analysis of the coated specimen N123 was done by FESEM and the composition scanning (EDX) images shown in Fig. 11. The FESEM was conducted on the examined nanocomposite coating on which the surface scanning was performed, and EDX provides the results of the examination of the coated steel N123. The findings of surface scanning show a homogeneous distribution of elements in the structure.

Economic losses due to metallic corrosion reached billions of dollars per year worldwide^44–46^. Epoxy is considered to be the most conventional and superior coating because of its good adhesion properties, excellent scratch hardness, etc.^27^. Nevertheless, epoxy coatings may fail under severe environmental conditions for prolonged exposure^28^. Poor adhesions of the coating can cause not only delamination of the coating layers but also the corrosion of the steel beneath the polymeric coating^47,48^. The alumina thin films coating have high mechanical properties and corrosion resistance, so, they have been applied in many industrial fields such as gas diffusion barriers, surface passivation, antireflection layers, etc.^49^. Producing protective coating with the inclusion of Al_2_O_3_ micro/nanoparticles into epoxy coating could have a great potential for commercial applications using metallic surfaces^50^. Nanocoatings to metallic surfaces can be used in equipment design thus lowering the maintenance and the working cost^51^.

## Conclusions

In this study, the tensile, hardne+ss, and barrier properties of steel lined with multilayered epoxy filled with Al_2_O_3_ particles in micron and nano size were investigated. The results showed that barrier resistance against salt and citric acid media were significantly enhanced by adding either nano or micron-sized Al_2_O_3_ particles to epoxy coatings. Nanocomposite coating has higher mechanical and barrier properties than microcomposite coatings. A maximum improvement of 48.4% and 90.48% was attained with epoxy liner filled with 3 wt% Al_2_O_3_ under dry and wet conditions, respectively. However, a maximum enhancement in tensile strength of 6.92% and 4.33% was obtained with epoxy liner filled with 1 wt% Al_2_O_3_ nanoparticles under dry and seawater conditions, respectively. With further increase in Al_2_O_3_ micro/nanoparticles to the epoxy liner, the uptake percentage of salt and citric acid solution increased.

## Data Availability

The datasets used during the current study available from the corresponding author on reasonable request.
